# CircRNA circ_POLA2 Promotes Cervical Squamous Cell Carcinoma Progression via Regulating miR-326/*GNB1*

**DOI:** 10.3389/fonc.2020.00959

**Published:** 2020-07-16

**Authors:** Yuan Cao, Juan Li, Yanyan Jia, Ruitao Zhang, Huirong Shi

**Affiliations:** ^1^Department of Gynaecology, The First Affiliated Hospital of Zhengzhou University, Zhengzhou, China; ^2^Key Laboratory of Clinical Medicine, The First Affiliated Hospital of Zhengzhou University, Zhengzhou, China

**Keywords:** cervical squamous cell carcinoma, circ_POLA2, *GNB1*, invasion, miR-326, proliferation *GNB1*

## Abstract

Circular RNAs (circRNAs) are a group of non-coding RNAs that have an essential function in the development and progression of various cancers. The expression pattern and function of circRNA in cervical squamous cell carcinoma (CESC) are not fully understood. In the present study, we aimed to investigate the expression profiles and regulation mechanism of circRNA circ_POLA2 in CESC. Circ_POLA2 was highly expressed in CESC tissues and positively correlated with poor prognosis in CESC patients. Knockdown of circ_POLA2 using shRNA inhibited cervical cancer cell proliferation, migration, and invasion both *in vitro* and *in vivo*. Mechanistically, circ_POLA2 could sponge endogenous microRNA-326 (miR-326) and inhibit its expression. Furthermore, miR-326 negatively regulated G protein subunit beta 1 (*GNB1*) by targeting its 3′-UTR. Intriguingly, we found that *GNB1* was overexpressed and associated with poor prognosis in CESC patients. Overexpression of *GNB1* could antagonize the inhibitory effect of miR-326 on cervical cancer cell proliferation, migration, and invasion. In addition, we demonstrated that circ_POLA2/miR-326/*GNB1* axis regulated ERK signaling. In conclusion, circ_POLA2 promotes cervical squamous cell carcinoma development and progression via regulating the miR-326/*GNB1* axis, which might serve as a novel therapeutic target for CESC patients.

## Introduction

Cervical squamous cell carcinoma (CESC) is one of the most common malignancies in women worldwide (1). CESC ranks as the fourth leading cause of cancer-related death in females, and most patients are diagnosed at a later stage with invasive carcinoma (2, 3). The incidence rate of CESC has dropped significantly as a result of early stage screening and human papillomavirus (HPV) vaccination in recent years (4). However, the survival rate of CESC patients diagnosed at advanced stages with lymphatic metastasis and distant metastasis is still very low (5). The molecular mechanism of CESC development and progression is complicated and not fully depicted. Thus, it is of great significance to explore the functional molecular and regulation mechanisms of CESC tumorigenesis.

Circular RNAs (circRNAs) are a group of non-coding RNAs with no protein translation (6). Different from other non-coding RNAs such as microRNA and long non-coding RNA (lncRNA), circRNAs form a closed loop with more stable construction (7). Mounting evidence has demonstrated that circRNAs play important roles in the tumorigenesis and metastasis (8, 9). CircRNAs exert their functions via different mechanisms, such as acting as competing endogenous RNA (ceRNA) by sponging miRNA, splicing pre-mRNAs, or blocking protein activity by decoying target protein (7, 10–12). In hepatocellular carcinoma (HCC), circRNA cSMARCA5 was demonstrated to inhibit HCC development via sponging miR-181-5p and miR-17-3p (13). CircRNA circGFRA1 functioned as a miR-34a sponge through regulation of GFRA1 expression in triple negative breast cancer (14). CircRNAs are also discovered as novel biomarkers in cervical cancer via microarray screening (15). CircRNA circ_0023404 was found highly expressed in cervical cancer that was correlated with poor prognosis of cervical cancer patients. Circ_0023404 might sponge miR-136 and regulate TFCP2 expression. Knocking down circ_0023404 suppressed cell proliferation, migration, and metastasis of cervical cancer cells (16). Tian et al. reported that circ_001445 was decreased in cervical cancer cells and it interacted with miR-620 to regulate cervical cancer development and progression (17). More circRNAs have been discovered to be involved in cervical cancer progression, and their function remains largely unknown (15).

MiRNAs are a group of small non-coding RNAs with ~22 nucleotide that posttranscriptionally regulate gene expression by binding to the 3′-UTR of their target mRNAs (18). Accumulating studies have found that miRNAs play critical roles in the tumorigenesis of most human malignancies, including cervical cancer (19, 20). The circRNA–miRNA–mRNA network plays a critical regulatory role in the pathology of cervical cancer (21). For instance, hsa_circ_0000515 promoted cervical cancer progression via sponging miR-326 and upregulating ETS transcription factor ELK1 (22). G protein subunit beta 1 (*GNB1*), which is a novel transduction protein, has been demonstrated to play a critical role in breast cancer (23). However, the function of *GNB1* in CESC is not clear.

A previous study reported that circ_POLA2 (hsa_circ_0022812) was overexpressed in CESC tissues (24). However, the functional role of circ_POLA2 in CESC and its regulatory mechanisms are still unknown. In the present study, we further confirmed the high expression of circ_POLA2 in a relatively large-scale CESC cohort. In addition, high circ_POLA2 expression predicts poor clinical outcomes in osteosarcoma. Functional experiments indicated that circ_POLA2 regulated cervical cancer cell proliferation, migration, and invasion via sponging miR-326 and regulating expression of *GNB1*. Overexpression of *GNB1* could abrogate the inhibitory function of miR-326 in cervical cancer cell growth and metastasis. These findings provide a valuable potential biomarker and therapeutic target for treatment of CESC via regulating the circ_POLA2/miR-326/*GNB1* network.

## Materials and Methods

### Patient Specimens

Cervical squamous cell carcinoma (CESC) tissues and adjacent normal control tissues (90 pairs) were collected from patients under surgery at the First Affiliated Hospital of Zhengzhou University (ZZU). Informed consent was obtained from each patient. The study was approved by the Research Ethics Committee of the First Affiliated Hospital of Zhengzhou University and conducted in compliance with the principles of the Declaration of Helsinki.

### Cell Culture

Human cervical cancer cell lines (Hela, SW756, CaSki, C-33a, and SiHa) and the control cervical epithelial cell line CerEpiC were purchased from ATCC (Manassas, VA, USA) or Cell Bank of Chinese Academy of Sciences (Shanghai, China) and maintained in the laboratory. Cells were cultured with Dulbecco's Modified Eagle's Medium (DMEM, Invitrogen, Carlsbad, CA, USA) containing 10% fetal bovine serum (FBS, Gibco, Gaithersburg, MD, USA), 1% penicillin, and streptomycin (Life Technologies, Grand Island, NY, USA) in a 5% CO_2_ incubator at 37°C.

### Transfection

ShRNA vector targeting circ_POLA2 and control plasmid, siRNA targeting *GNB1*, and a scramble negative control were obtained from GenePharma (Shanghai, China). MiR-326 mimics, inhibitor, and relative negative controls were purchased from RiboBio Technology (Guangzhou, China). Full-length circ_POLA2 and *GNB1* was amplified and cloned into pcDNA3.1 vector to generate overexpression vector pcDNA3.1-circ_POLA2 and pcDNA3.1-*GNB1*. Transfection was performed using Lipofectamine 3000 (Thermo Fisher Scientific, Waltham, MA, USA). Lentivirus containing control or circ_POLA2-targeting shRNA was used to infect cells, and circ_POLA2 stable-knockdown cell lines were obtained after puromycin selection. The knockdown or overexpression efficiency was evaluated by real-time quantitative polymerase chain reaction (RT-qPCR).

### RT-qPCR

Total RNA was extracted from tissue samples or cells using Trizol (Invitrogen, Carlsbad, CA, USA) and reversed transcribed into cDNA using high-capacity cDNA Reverse Transcription Kit (Applied Biosystems, Foster City, CA, USA). RT-qPCR was performed using SYBR Green master mix (Applied Biosystems, Foster City, CA, USA). Housekeeping genes *GAPDH* and *U6* were used as internal controls. The PCR primers used were listed in Supplementary Tables 1, 2.

### Cell Growth Assay

Cell growth was evaluated by Cell Counting Kit-8 (CCK-8) assay (Dojindo, Kumamoto, Japan), colony formation assay, and 5′-ethynyl-20-deoxyuridine (EdU) staining assay as described previously (25).

### Cell Migration and Invasion Assay

Cell migration was assessed by wound-healing assay and cell invasion was examined by transwell assay using 24-well invasion chambers coated with Matrigel (Corning, Corning, NY, USA) as previously described (26).

### Western Blot

Total protein was extracted from tissue samples or cultured cells using cell lysis buffer (Cell Signaling Technology, Danvers, MA, USA) and the protein concentration was determined using a bicinchoninic acid (BCA) kit (Thermo Scientific Pierce Protein Biology, Hanover Park, IL, USA). Equal amount of protein was separated by sodium dodecyl sulfate-polyacrylamide gel electrophoresis (SDS-PAGE) and transferred onto a nitrocellulose membrane for western blot. The antibodies used in the experiments are listed in Supplementary Table 3.

### Immunohistochemical Staining and *in situ* Hybridization

Cervical squamous cell carcinoma tissues and adjacent control tissues were used to construct a tissue microarray (TMA). Immunohistochemical staining of *GNB1* and *in situ* hybridization (ISH) of circ_POLA2 were performed as previously described (27, 28). The expression level was classified into five scale scores based on the staining intensity. For tissue microarray analysis, sections were semiquantitatively scored for the circRNA or *GNB1* staining patterns as follows: the staining extent in each core was scored as 1+ (<25% staining of tumor cells), 2+ (25–50% staining of tumor cells), 3+ (50–75% staining of tumor cells), or 4+ (> 75% staining of tumor cells). Additionally, the staining intensity was quantified as 0 (negative), 1+ (weak), 2+ (intermediate), or 3+ (strong). The final immunoreaction score was obtained by multiplying the intensity and extension values (range 0–12) and the samples were grouped as 1+ (score 0), 2+ (score 1–2), 3+ (score 3–4), 4+ (score 6–8), and 5+ (score 9–12) staining. Meanwhile, for statistical purposes, scores of 4+ and 5+ were defined as high expression and the other final scores were considered as low expression. Categorizing circRNA or *GNB1* staining was completed by two independent investigators (Yuan Cao and Ruitao Zhang). Mouse SW756 xenograft tumor tissues were fixed and paraffin embedded and then cut into sections. Hematoxylin and eosin (H&E) staining was performed and cell proliferation was evaluated by Ki-67 staining (Ki-67, D2H10, Cell Signaling Technology, Danvers, MA, USA).

### Xenograft Tumor Model

Xenograft tumor experiment was performed using male nude mice (Vital River Laboratory, Beijing, China). 2 × 10^6^ SW756 cervical cancer cells with stable knockdown circ_POLA2 or control cells were injected subcutaneously into the right flank of nude mice. Tumor growth was monitored every week and tumor size was measured by a vernier caliper to calculate the volume (length × width^2^)/2. Mice were euthanized after 5 weeks and tumors were collected for analysis. For *in vivo* metastasis study, 1 × 10^6^ cervical cancer cells with stable knockdown circ_POLA2 or control cells were injected into nude mice via the tail vein. The mice were sacrificed 7 weeks later. Lungs were harvested, weighed, and then sectioned and stained by H&E. The animal experiment was approved by the Experimental Animal Ethics Committee of Zhengzhou University.

### Luciferase Reporter Assay

Luciferase reporter vectors containing wild-type (WT) or mutated circ_POLA2 and vectors containing WT or mutated 3′-UTR of *GNB1* were constructed using the backbone vector pGL3-Luc. SW756 cells were transfected with reporter vector, together with miR-326 mimics or negative control. Relative luciferase activity (firefly luciferase vs. *Renilla* signal) was analyzed with a dual-luciferase reporter assay kit (Promega, San Luis Obispo, CA, USA) 48 h later.

### Bioinformatics Analysis of Microarray and Target Prediction

RNA-seq data of TCGA were downloaded and log transformed before analysis. Putative miRNA-binding sites in the circ_POLA2 or *GNB1* sequence were predicted using StarBase V2.0 (http://starbase.sysu.edu.cn/) or CircInteractome (https://circinteractome.nia.nih.gov/) as officially recommended steps.

### Statistical Analysis

Data are represented as the means ± SD. Statistical analysis was performed in GraphPad Prism, version 8.0 (GraphPad Prism Software, GraphPad, San Diego, CA, USA), using the two-tailed Student *t*-test for comparison of two groups and one-way ANOVA followed by Tukey *post hoc* test for two or more groups. The analysis of correlation between factors was performed by Pearson's correlation coefficient rank test. The Kaplan–Meier and log-rank test method was performed to determine survival rate. A *p* < 0.05 was considered to be statistically significant.

## Results

### Circ_POLA2 Is Upregulated in CESC and High Expression of circ_POLA2 Predicts Poor Prognosis in CESC Patients

**Figure 1:**
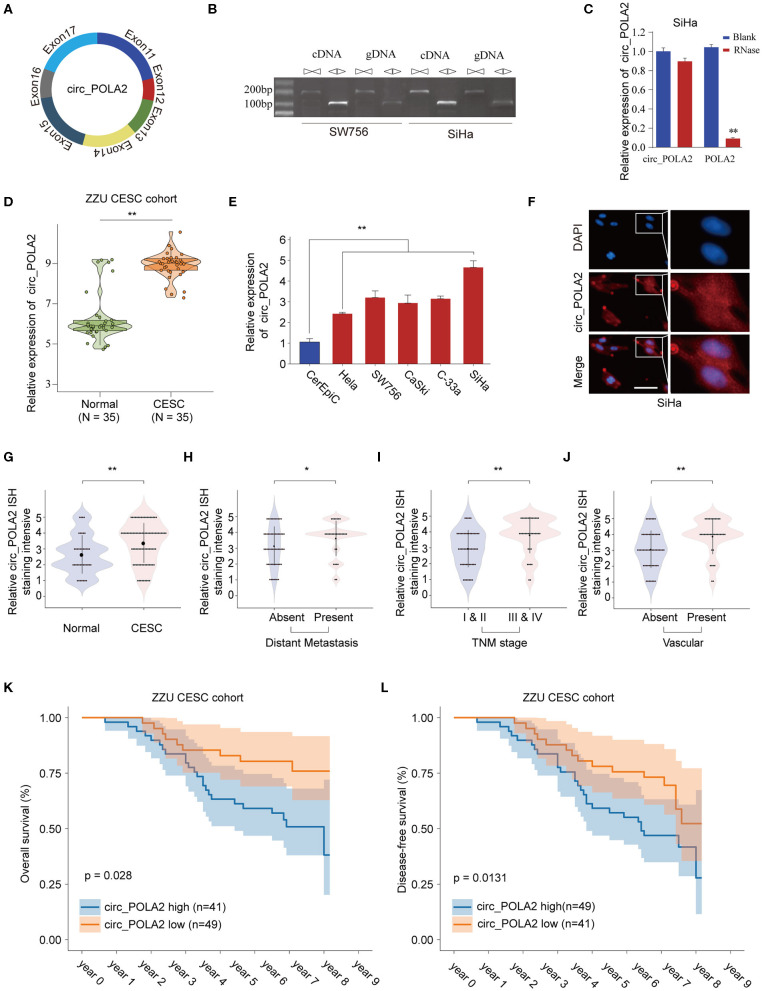
Expression profile of circ_POLA2 in CESC and the correlation of circ_POLA2 expression with CESC progression and prognosis. **(A)** Diagram of circ_POLA2 consisting of Exon11-17. **(B)** RT-PCR assay with divergent or convergent primers indicating the existence of circ_POLA2 in the SW756 and SiHa cell line. **(C)** RT-PCR analysis of circ_POLA2, linear POLA2, and β-actin in SiHa cells treated with RNase R. **(D)** Expression levels of circ_POLA2 in 35 paired CESC tissues and adjacent normal tissues from ZZU CESC cohort analyzed by qRT-PCR. **(E)** Expression levels of circ_POLA2 in control human cervical epithelial cell (CerEpic) and cervical cancer cell lines (Hela, SW756, CaSki, C-33a, and SiHa) analyzed by qRT-PCR. **(F)** Detection of colocalization of circ_POLA2 expression in cytoplasm by RNA FISH assay (magnification, ×400). Nuclei were stained in blue (DAPI), and circ_POLA2 was stained in red. *In situ* hybridization staining of circ_POLA2 was performed on 90 paired CESC and adjacent normal tissue sections. The staining intensity of circ_POLA2 in normal or CESC tissues **(G)**, in CESC tissues with or without distant metastasis **(H)**, in CESC tissues with different TNM stages **(I)**, or in CESC tissues with or without vascular invasion **(J)** was analyzed. **(K,L)** Kaplan–Meier analysis of overall survival (OS) and disease-free survival (DFS) in 90 CESC patients with low or high circ_POLA2 expression from ZZU CESC cohort. ^*^p < 0.05; ^**^p < 0.01.

Circ_POLA2 is produced with back splicing of seven scrambled exons (exons 11–17, Figure 1A). Circ_POLA2 was first confirmed via PCR in both cDNA and genome DNA (gDNA), and the results indicated that PCR with gDNA yielded no products while cDNA yielded completely consistent sequences for Circ_POLA2 (Figure 1B). Moreover, resistance to RNase exonuclease confirmed that Circ_POLA2 was indeed circular (Figure 1C). Subsequently, expression levels of circ_POLA2 in 35 paired CESC tissues and adjacent normal tissues from the ZZU CESC cohort were assessed by RT-qPCR. The results showed that POLA2 was significantly upregulated in CESC tissues in comparison with that in normal control tissues (Figure 1D). Cervical cancer cell lines (Hela, SW756, CaSki, C-33a, and SiHa) also had higher levels of circ_POLA2 than that in human cervical epithelial cell line CerEpiC (Figure 1E). Localization of circ_POLA2 detected by fluorescence *in situ* hybridization (FISH) in Siha cells showed that it was localized in the cytoplasm (Figure 1F).

To further evaluate the expression of circ_POLA2 in CESC, we performed ISH staining of circ_POLA2 in 90 paired CESC and adjacent normal tissues (Supplementary Figure 1). CESC tissues had enhanced circ_POLA2 staining intensity compared to that in normal tissues (Figure 1G). Meanwhile, higher circ_POLA2 expression was positively associated with distant metastasis, advanced TNM stage, and presence of vascular invasion (Figures 1H–J). Kaplan–Meier analysis was conducted to investigate the relationship between circ_POLA2 expression and prognosis of CESC patients in ZZU cohort. As shown in Figures 1K,L, CESC patients with high expression of circ_POLA2 exhibited significantly worse overall survival (OS) and disease-free survival (DFS) in comparison with that in CESC patients with low expression of circ_POLA2 in ZZU CESC cohort. These findings suggest that circ_POLA2 is overexpressed in CESC and predicts poor prognosis in CESC patients.

### Silencing circ_POLA2 Inhibits Cervical Cancer Cell Proliferation, Invasion, and Migration

**Figure 2:**
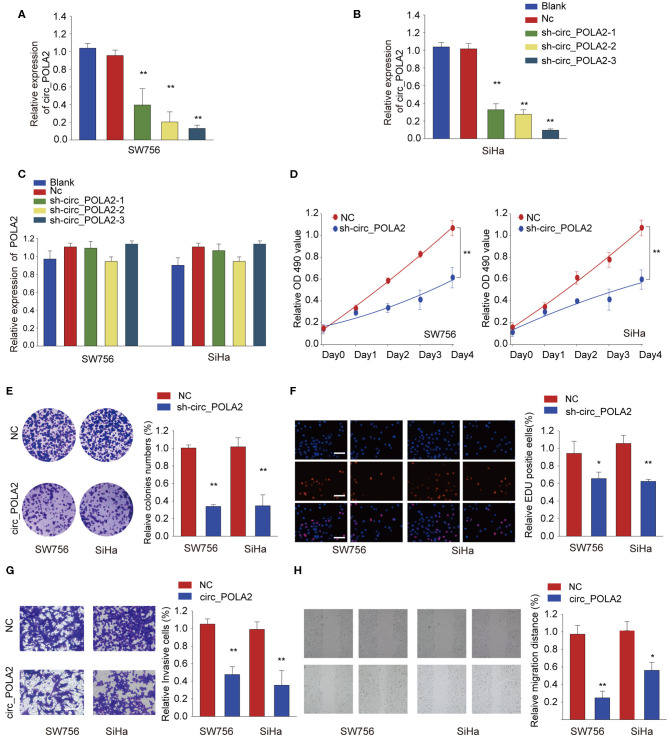
Silencing circ_POLA2 inhibits cervical cancer cell proliferation, invasion, and migration. Cervical cancer cell line SW756 or SiHa was left untreated (Blank), or transfected with negative control (NC) or shRNA targeting circ_POLA2 (sh-circ_POLA2-1/2/3). The relative expression levels of **(A,B)** circ_POLA2 or **(C)** POLA2 mRNA were analyzed by qRT-PCR 48 h later. **(D–F)** SW756 or SiHa cells were transfected with NC or sh-circ_POLA2-3. Cell proliferation was assessed by CCK-8 assay **(D)**, colony formation assay **(E)**, and EDU staining **(F,G)** Invasive capability of SW756 or SiHa cells transfected with NC or sh-circ_POLA2 was assessed by transwell assay. **(H)** The migration capability of SW756 or SiHa cells transfected with NC or sh-circ_POLA2 was assessed by wound-healing assay. Scale bars, 50 μm. ^*^p < 0.05; ^**^p < 0.01.

To explore the function of circ_POLA2, a loss-of-function assay was performed using shRNA targeting circ_POLA2. While sh-circ_POLA2 could specifically silence the expression of circ_POLA2 in SW756 or SiHa cells, the relative expression of POLA2 mRNA remained the same (Figures 2A–C). The most efficient sh-circ_POLA2-3 was selected to evaluate the biological function of circ_POLA2 knockdown. As shown in Figures 2D–F, knockdown of circ_POLA2 significantly dampened cell proliferation, colony formation, and DNA synthesis in SW756 or SiHa cells. Transwell assay revealed that knockdown of circ_POLA2 inhibited cell invasion of SW756 or SiHa cells (Figure 2G). Silencing circ_POLA2 also suppressed cell migration in SW756 or SiHa cells, as demonstrated by wound-healing assay (Figure 2H). Collectively, silencing circ_POLA2 inhibits cervical cancer cell proliferation, invasion, and migration.

### Silencing circ_POLA2 Suppresses Cervical Cancer Development *in vivo*

**Figure 3:**
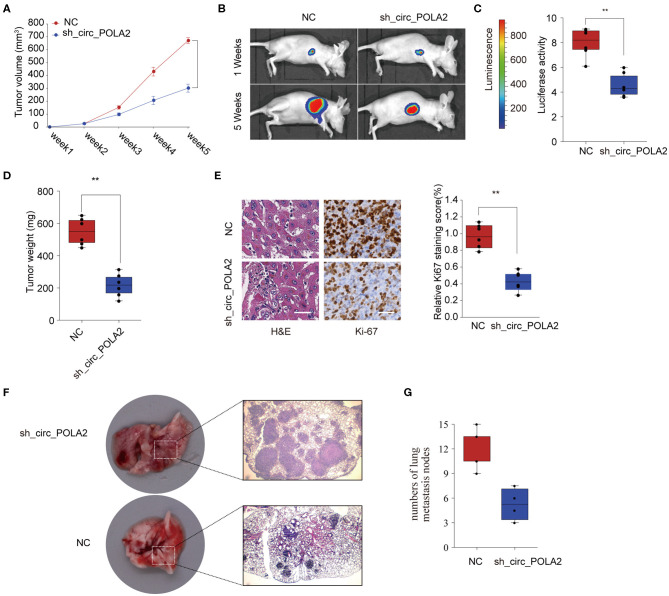
Silencing circ_POLA2 suppresses cervical cancer development *in vivo*. Cervical cancer cells SW756 stably transfected with negative control (NC) or sh-circ_POLA2 were intravenously injected into nude mice. **(A)** Tumor volume was measured and determined at indicated time points. **(B)** Representative bioluminescent photos were recorded at 1 week or 5 weeks post injection. **(C)** Relative luciferase activity was analyzed in the NC or sh-circ_POLA2 group. **(D)** Tumors from the NC or sh-circ_POLA2 group were isolated from nude mice and tumor weight was examined. **(E)** Representative H&E staining and immunohistochemical staining image of Ki-67 were acquired on tumor sections from the NC or sh-circ_POLA2 group (left panel). Relative Ki-67 staining intensity in the NC or sh-circ_POLA2 group was analyzed (right panel). **(F)** Macroscopic appearances and representative H&E–stained images of lung metastasis are shown. **(G)** The number of tumor nodules was evaluated and analyzed. Scale bars, 100 μm. ^**^p < 0.01.

To further investigate the function of circ_POLA2 *in vivo*, SW756 cells with stably circ_POLA2 knockdown or control cells were implanted into nude mice and tumor volume was monitored. Knockdown of circ_POLA2 significantly suppressed cervical cancer development (Figure 3A). Tumors from the sh-circ_POLA2 group had much smaller tumor size and lower tumor weight than those in the NC group (Figures 3B–D). Furthermore, we found that knockdown of circ_POLA2 markedly decreased the expression of proliferation marker Ki-67 in tumor sections, as demonstrated by immunohistochemical staining (Figure 3E). Furthermore, circ_POLA2 silencing reduced the numbers and sizes of metastatic lung tumors at 40 days post inoculation (Figures 3F,G). These results suggest that silencing circ_POLA2 suppresses cervical cancer proliferation and metastasis *in vivo*.

### Circ_POLA2 Is an miRNA Sponge Interacting With miR-326

**Figure 4:**
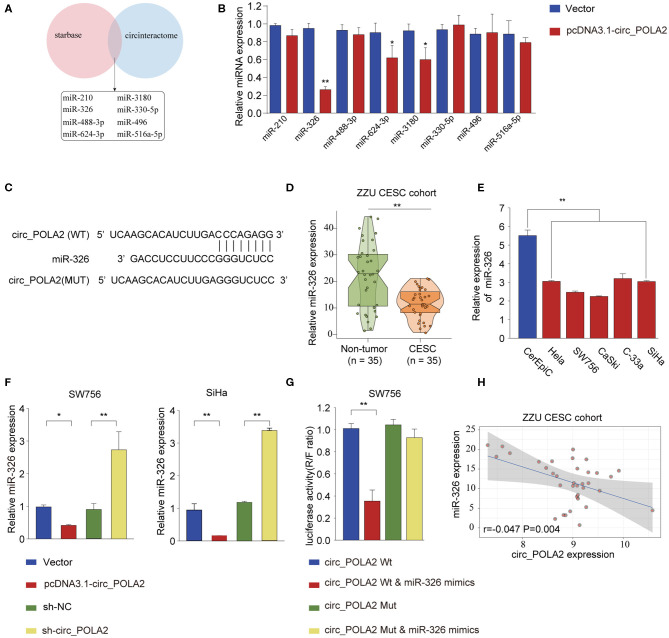
Circ_POLA2 is a miRNA sponge interacting with miR-326. **(A)** Bioinformatics analysis was performed using StarBase (http://starbase.sysu.edu.cn/index.php) and CircInteractome (https://circinteractome.nia.nih.gov/) to predict the potential miRNAs interacting with circ_POLA2. **(B)** SW756 cells were transfected with control vector or pcDNA3.1-circ_POLA2. The relative expression of miRNA was analyzed by qPCR 48 h later. **(C)** Putative binding sequences between circ_POLA2 and miR-326. **(D)** The expression levels of miR-326 in 35 paired CESC tissues and non-tumor tissues were analyzed by qRT-PCR. **(E)** The expression levels of miR-326 in control human cervical epithelial cell (CerEpic) and cervical cancer cell lines (Hela, SW756, CaSki, C-33a, and SiHa) were analyzed by qRT-PCR. **(F)** SW756 or SiHa cells were transfected with negative control (NC), pcDNA3.1-circ_POLA2, sh-NC, or sh-circ_POLA2. The relative expression of miR-326 was analyzed by qRT-PCR 48 h later. **(G)** SW756 cells were transfected with luciferase reporter plasmid containing WT circ_POLA2 or mutated circ_POLA2, together with or without miR-326 mimics. The relative luciferase activity in SW756 cells was analyzed 48 h later. **(H)** Pearson analysis of the correlation between miR-326 expression and circ_POLA2 expression in 35 paired CESC tissues and non-tumor tissues. ^*^p < 0.05; ^**^ *p* < 0.01.

Accumulating evidence has shown that circ_RNAs could act as miRNA sponges to exert their function (12). We performed bioinformatics analysis using starBase (http://starbase.sysu.edu.cn/index.php) and CircInteractome (https://circinteractome.nia.nih.gov/) to predict the potential miRNAs interacting with circ_POLA2. As shown in Figure 4A, eight miRNAs (miR-210, miR-326, miR-488-3p, miR-624-3p, miR-3180, miR-330-5p, miR-496, and miR-516a-5p) were predicted by both tools. SW756 cells were transfected with control vector or pcDNA3.1-circ_POLA2 to overexpress circ_POLA2. The results showed that overexpression of circ_POLA2 significantly inhibited the relative expression of miR-326, miR-624-3p, and miR-3180 (Figure 4B). Here we focused on the most downregulated miR-326 in this study. Bioinformatics analysis found that miR-326 had the complementary binding sequences with circ_POLA2 (Figure 4C). In contrast to the expression of circ_POLA2, CESC tissues or cervical cancer cell lines had significantly lower levels of miR-326 than those in non-tumor tissues or control cell line (Figures 4D,E). Moreover, overexpression of circ_POLA2 inhibited miR-326 expression while knockdown of circ_POLA2 enhanced miR-326 expression in SW756 cells (Figure 4F). A luciferase reporter assay was conducted and the results showed there was a direct interaction between miR-326 and WT circ_POLA2 sequences, but not with mutated circ_POLA2 sequences (Figure 4G). Pearson correlation analysis showed that the expression of miR-326 was negatively associated with circ_POLA2 expression (Figure 4H, *p* = 0.0013). Collectively, these findings indicate circ_POLA2 is a miRNA sponge interacting with miR-326.

### Circ_POLA2 Promotes Cervical Cancer Cell Proliferation and Invasion via Sponging miR-326

**Figure 5:**
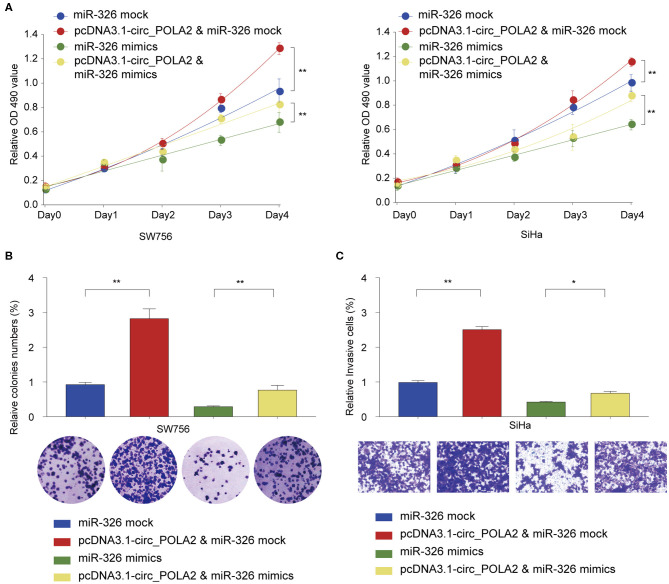
Circ_POLA2 promotes cervical cancer cell proliferation and invasion via sponging miR-326. **(A)** SW756 or SiHa cells were transfected with miRNA mock, pcDNA3.1-circ_POLA2 + miRNA mock, miR-326 mimics, or pcDNA3.1-circ_POLA2 + miR-326 mimics. Cell growth was assessed at the indicated time points using CCK-8. **(B,C)** SW756 cells were transfected with miRNA mock, pcDNA3.1-circ_POLA2 + miRNA mock, miR-326 mimics, or pcDNA3.1-circ_POLA2 + miR-326 mimics. **(B)** Cell proliferation and **(C)** cell invasion were analyzed by colony formation assay and transwell assay, respectively. ^*^p < 0.05; ^**^p < 0.01.

We further tested the functional relationship between circ_POLA2 and miR-326. SW756 or SiHa cells were transfected with circ_POLA2 overexpression vector, miR-326 mimics, pcDNA3.1-circ_POLA2+miR-326 mimics, or relative control. Cell proliferation assay revealed that overexpression of circ_POLA2 promoted cervical cancer proliferation, while miR-326 mimics inhibited cell proliferation in SW756 or SiHa cells (Figure 5A). However, miR-326 overexpression antagonized the function of circ_POLA2 in regulating cell proliferation (Figure 5A). Furthermore, we demonstrated that the enhancement of cell colony formation and cell invasion in SW756 cells transfected with pcDNA3.1-circ_POLA2 was abrogated by miR-326 overexpression (Figures 5B,C). Thus, circ_POLA2 promotes cervical cancer cell proliferation and invasion via sponging miR-326.

### MiR-326 Negatively Regulates *GNB1* Expression via Targeting Its 3′-UTR

**Figure 6:**
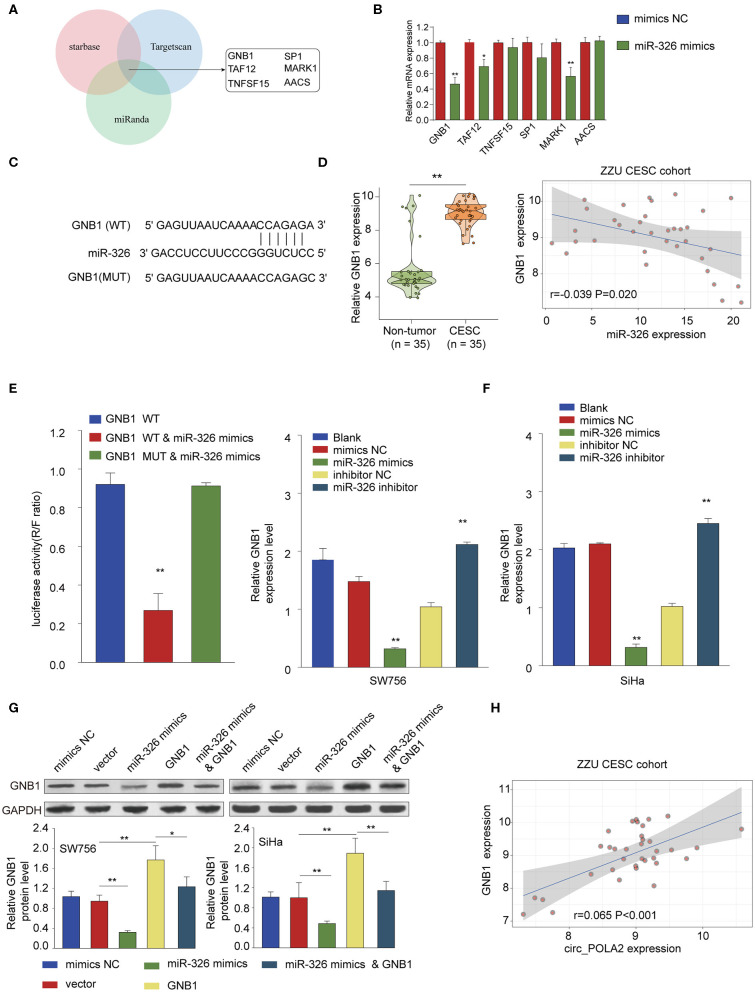
MiR-326 negatively regulates *GNB1* expression via targeting its 3′-UTR. **(A)** Bioinformatics analysis was performed using multiple online tools (StarBase, miRanda, and TargetScan) to predict the potential targets of miR-326. **(B)** SW756 cells were transfected with miR-326 mimics or negative control. The relative expression of potential targets was analyzed by qPCR 48 h later. **(C)** Putative binding sequences between WT *GNB1* and miR-326. **(D)** Relative *GNB1* expression and Pearson analysis of the correlation between *GNB1* expression and miR-326 expression in 35 paired CESC tissues and non-tumor tissues. **(E)** SW756 cells were transfected with luciferase reporter plasmid containing WT 3′-UTR of *GNB1* or mutated 3′-UTR of *GNB1*, together with or without miR-326 mimics. The relative luciferase activity in SW756 cells was analyzed 48 h later. **(F)** SW756 or SiHa cells were left untreated (Blank), or transfected with negative control (mimics NC or inhibitor NC), miR-326 mimics, or miR-326 inhibitor. The relative expression of *GNB1* was analyzed by qRT-PCR 48 h later. **(G)** SW756 or SiHa cells were transfected with negative control (mimics NC), pcDNA3.1 vector (Vector), miR-326 mimics, pcDNA3.1-*GNB1* (*GNB1*), or miR-326 mimics + pcDNA3.1-*GNB1*. The relative protein expression of *GNB1* was analyzed by western blot 48 h later. GAPDH was used as a loading control. **(H)** Pearson analysis the correlation between *GNB1* expression and circ_POLA2 expression in 35 paired CESC tissues and non-tumor tissues. ^*^p < 0.05; ^**^p < 0.01.

Further bioinformatics analysis using multiple online tools (StarBase, miRanda, and TargetScan) was performed to explore the downstream target of circ_POLA2 and miR-326. As shown in Figure 6A, six targets (*GNB1, SP1, TAF12, MARK1, TNFSF15*, and *AACS*) were predicted as the potential targets of miR-326. Further, we verified the interaction between miR-326 and potential targets via transfecting SW756 cells with miR-326 mimics or negative control. Overexpression of miR-326 significantly inhibited the expression of *GNB1, TAF12*, or *MARK1* (Figure 6B). As *GNB1* mutations were found in various tumors and associated with hematological transformation (29), we further investigated the functional role of *GNB1* in CESC. *GNB1* was predicted as a direct target of miR-326 and miR-326 had the complementary binding sequences targeting the 3′-UTR of *GNB1* (Figure 6C). *GNB1* expression was negatively correlated with the expression of miR-326 in CESC tissues (Figure 6D). In addition, miR-326 mimics inhibited the luciferase activity in SW756 cells with reporter vector containing WT 3′-UTR of *GNB1* (Figure 6E). Overexpression of miR-326 decreased *GNB1* expression while inhibition of miR-326 enhanced *GNB1* mRNA and protein expression in SW756 or SiHa cells (Figures 6F,G). Intriguingly, we found that *GNB1* expression was positively associated with the expression of circ_POLA2 in CESC tissues (Figure 6H).

### *GNB1* Is Overexpressed in CESC and High Expression of *GNB1* Predicts Poor Prognosis in CESC Patients

**Figure 7:**
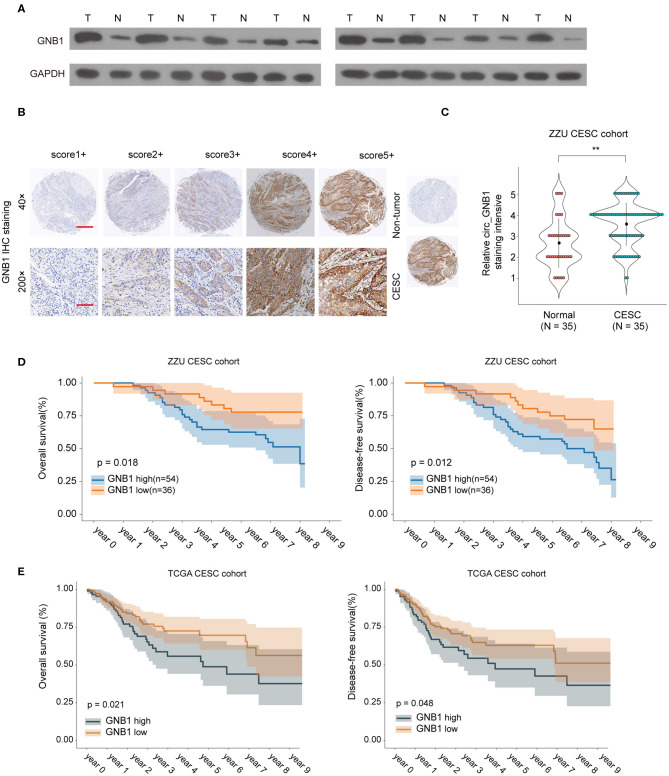
The expression pattern of *GNB1* in CESC and the correlation of *GNB1* expression with CESC prognosis. **(A)** The protein expression of *GNB1* in CESC tissues or adjacent normal tissues was analyzed by western blot. **(B)** Representative images of *GNB1* immunohistochemical (IHC) staining with different staining intensity scores. **(C)** Representative IHC staining image of *GNB1* and the distribution of *GNB1* IHC staining scores in CESC tissues or non-tumor normal tissues. **(D)** Kaplan–Meier analysis of OS and DFS in 90 CESC patients with low or high *GNB1* expression from the ZZU CESC cohort. **(E)** Kaplan–Meier analysis of OS and DFS in CESC patients with low or high *GNB1* expression from the TCGA CESC cohort. Scale bars, 100 μm for 200×, 500 μm for 40×. ^**^p < 0.01.

To further investigate the expression pattern and function of *GNB1* in CESC, western blot was performed and the protein expression of *GNB1* in CESC tissues was much higher than that in adjacent normal tissues (Figure 7A). The enhanced expression of *GNB1* in CESC tissues was also confirmed by immunohistochemical staining of *GNB1* in CESC tissues or non-tumor tissues (Figures 7B,C). Kaplan–Meier analysis of OS and DFS demonstrated that CESC patients with high *GNB1* expression had a poorer prognosis than patients with low *GNB1* expression in the ZZU CESC cohort and the TCGA CESC cohort (Figures 7D,E). Taken together, the results suggest that *GNB1* is overexpressed in CESC, and high expression of *GNB1* predicts poor prognosis in CESC patients.

### *GNB1* Overexpression Antagonizes the Inhibitory Effect of miR-326 on Cervical Cancer Cell Proliferation and Invasion via Regulating ERK Signaling

**Figure 8:**
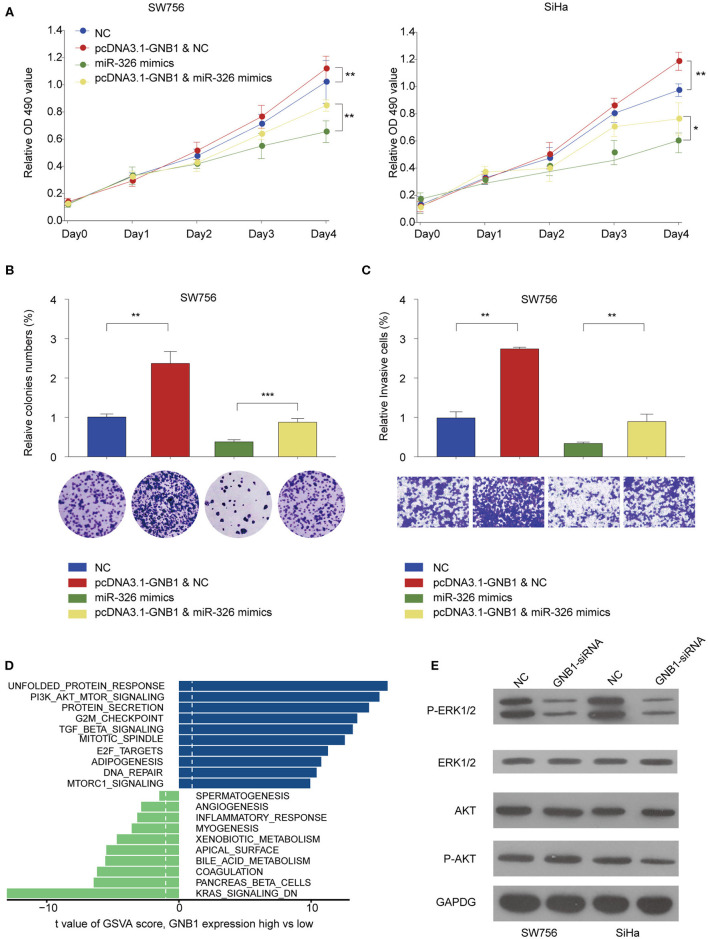
*GNB1* overexpression antagonizes the inhibitory effect of miR-326 on cervical cancer cell proliferation and invasion via regulating ERK signaling. **(A)** SW756 or SiHa cells were transfected with negative control (NC), pcDNA3.1-*GNB1*+NC, miR-326 mimics, or pcDNA3.1-*GNB1* + miR-326 mimics. Cell growth was assessed at the indicated time points using CCK-8. **(B,C)** SW756 cells were transfected with NC, pcDNA3.1-*GNB1* + NC, miR-326 mimics, or pcDNA3.1-*GNB1* + miR-326 mimics. **(B)** Cell proliferation and **(C)** cell invasion were analyzed by colony formation assay and transwell assay, respectively. **(D)** Gene Set Variation Analysis of signaling pathway enriched with low or high *GNB1* expression. **(E)** SW756 or SiHa cells were transfected with NC or GNB1-siRNA. ^*^P < 0.05; ^**^P < 0.01; ^***^P < 0.001.

The functional relationship between *GNB1* and miR-326 was evaluated *in vitro*. *GNB1* overexpression promoted cell proliferation and miR-326 mimics inhibited cell proliferation in SW756 or SiHa cells (Figure 8A). *GNB1* overexpression dampened the inhibitory effect of miR-326 on cervical cell proliferation, colony formation, and invasion (Figures 8A–C). The results indicate that *GNB1* acts as the functional downstream target of circ_POLA2/miR-326. To further elucidate the mechanism underlying the regulatory axis of circ_POLA2/miR-326/*GNB1* in CESC, Gene Set Variation Analysis (GSVA) was performed and multiple signaling pathways involved in the function of circ_POLA2/miR-326/*GNB1* were identified (Figure 8D). SW756 or SiHa cells were transfected with siRNA knocking down *GNB1*. Silencing *GNB1* markedly inhibited ERK phosphorylation while it did not affect the phosphorylation level of AKT (Figure 8E). The results indicated that *GNB1* might regulate cervical cancer cell proliferation and invasion via controlling ERK signaling.

## Discussion

Next-generation sequencing has identified numerous circRNAs and their function in tumorigenesis is undergoing extensively investigation (30, 31). DNA polymerase α2 accessory subunit (POLA2), a subunit of DNA polymerase α regulating DNA replication, was identified as a prognostic biomarker for ovarian cancer and gastric carcinoma (32, 33). In the current study, we identified a novel circRNA circ_POLA2 produced with back splicing of seven scrambled exons of POLA2. Circ_POLA2 functions as a ceRNA by sponging miR-326 and regulates the expression of *GNB1* in CESC.

Various circRNAs are involved in the initiation, progression, and metastasis of cervical cancer via different mechanisms (15). Sponging miRNA is one of the most common regulatory mechanisms of circRNA. Hsa_circRNA_101996 was found highly expressed in cervical cancer and could promote cancer development by sponging miRNA-8075, which targets TPX2 (34). Extensive profiling showed that circRNA-000284 promoted cell growth and invasion in cervical cancer via sponging miR-506 to suppress the expression of Snail-2 (35). We identified that the expression of circ_POLA2 was upregulated in CESC and high levels of circ_POLA2 were associated with poor prognosis. Functionally, we demonstrated that knockdown of circ_POLA2 suppressed cervical cancer cell proliferation, migration, and invasion both *in vitro* and *in vivo*. Thus, these findings suggest an oncogene role of circ_POLA2 in CESC.

Bioinformatics analysis predicted miR-326 interacted with circ_POLA2 and knockdown of circ_POLA2 increased miR-326 expression. Intriguingly, miR-326 expression was significantly lower in CESC compared with adjacent non-tumor tissues and its expression was negatively correlated with circ_POLA2 expression. MiR-326 was reported to function as a tumor suppressor in multiple cancers such as gastric cancer and non-small-cell lung cancer (36, 37). In cervical cancer, Tang et al. showed that circ_000515 could sponge miR-326 and promote cervical cancer development via enhancing ELK1 (22). In another study, miR-326 interacted with hsa_circ_0003998 in the progression of non-small-cell lung cancer (38). These studies indicate that expression of miR-326 is modulated by multiple circRNAs in cervical cancer. In contrast, circ_POLA2 might also regulate other miRNAs in the development of CESC, which needs further investigation.

There are also multiple-to-multiple relationships between miRNA and target genes in the regulatory networks (39). MiR-326 was reported to regulate cyclin D1 expression in non-small-cell lung cancer development (36). Proto-oncogenes *NOB1* and *phox2a* were also targeted by miR-326 in gastric cancer and lung cancer (37, 40). In this study, we predicted the targets of miR-326 by online databases and multiple genes were regulated by miR-326. *GNB1* was among the most inhibited target genes by miR-326 overexpression (data not shown). *GNB1* mutations were found in tumors resistant to different kinase inhibitors and also associated with hematological transformation (29). Here we identified that *GNB1* expression was positively correlated with circ_POLA2 expression and might function as an oncogene in CESC. In addition, GSVA analysis and *GNB1* knockdown also indicated that ERK signaling was regulated by circ_POLA2/miR-326/*GNB1* network.

In this study, we demonstrate that circ_POLA2 is highly expressed in CESC and associated with a poor prognosis in CESC patients. Circ_POLA2 regulates cervical cancer cell growth and metastasis via sponging miR-326. Furthermore, *GNB1* is a direct target of miR-326 and *GNB1* overexpression antagonizes the inhibitory effect of miR-326 on cervical cancer cell growth and metastasis. In summary, we demonstrate the potential regulatory role of circ_POLA2 in CESC development and metastasis sponging miR-326 and regulating *GNB1* expression. Our data provide a potential biomarker and therapeutic target for treatment of CESC via regulating circ_POLA2/miR-326/*GNB1* network.

## Data Availability Statement

Publicly available datasets were analyzed in this study. This data can be found here: The Cancer Genome Atlas (TCGA) https://www.cancer.gov/about-nci/organization/ccg/research/structural-genomics/tcga.

## Ethics Statement

The studies involving human participants were reviewed and approved by ethics committee of the first affiliated Hospital of Zhengzhou University. The patients/participants provided their written informed consent to participate in this study. The animal study was reviewed and approved by the experimental animal ethics committee of zhengzhou university.

## Author Contributions

All authors contributed to data analysis, drafting, or revising the article, gave final approval of the version to be published, and agree to be accountable for all aspects of the work.

## Conflict of Interest

The authors declare that the research was conducted in the absence of any commercial or financial relationships that could be construed as a potential conflict of interest.

## Supplementary Material

Supplementary Figure 1 Representative *in situ* hybridization staining of circ_POLA2 in 90 paired CESC and adjacent normal tissues with different staining intensity scores.
