# Supplementary material for: CircRNA circ_POLA2 Promotes Cervical Squamous Cell Carcinoma Progression via Regulating miR-326/GNB1
Source: Front Oncol. 2020 Jul 16;10:959. doi: 10.3389/fonc.2020.00959 (PMC7381119; doi:10.3389/fonc.2020.00959)
Supplement: Supplementary Figure 1 — Representative in situ hybridization staining of circ_POLA2 in 90 paired CESC and adjacent normal tissues with different staining intensity scores. [file Image_1.pdf]

Supplementary Figure S1

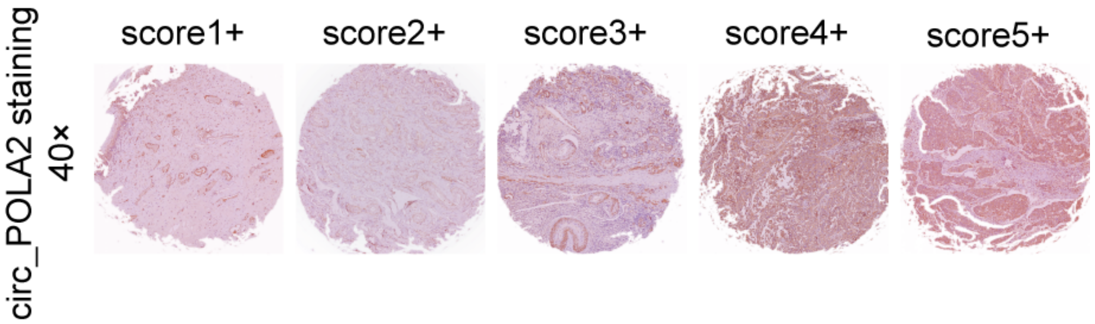

Representative circ_POLA2 in situ hybridization (ISH) staining patterns with different staining scores in CESC tissues.
