# Supplementary material for: CircRNA circ_POLA2 Promotes Cervical Squamous Cell Carcinoma Progression via Regulating miR-326/GNB1
Source: Front Oncol. 2020 Jul 16;10:959. doi: 10.3389/fonc.2020.00959 (PMC7381119; doi:10.3389/fonc.2020.00959)
Supplement: Supplementary file 2 [file Table_1.docx]

**Supplementary Table 1. Primer sequence used in this study**

| **siRNA** | **sense sequence** |
| --- | --- |
| miR-326 forward primer | 5'- AACAAGGACCTCCTTCCCGG -3' |
| miR-326 reverse primer | 5'- CAGTGCAGGGTCCGAGGT -3' |
| U6 forward primer | 5'-CCAGUUUACCUAACGCAAUTT-3' |
| U6 reverse primer | 5'-TTCACGAATTTGCGTGTCAT-3' |
| GAPDH forward primer | 5′-ACCAGGAAATGAGCTTGACA-3′ |
| GAPDH reverse primer | 5′-GACCACAGTCCATGCCATC-3′ |
| hsa_circ_POLA2 forward primer | 5′- TGAGCTTGTGAGTGAGTGGT -3′ |
| hsa_circ_POLA2 reverse primer | 5′- GCAAGGAGAATGGCGAGATG -3′ |
| GNB1 forward primer | 5′- TCACAAACAACATCGACCCAG -3′ |
| GNB1 reverse primer | 5′- CGAGGCACTGACGAGAAGC -3′ |

**Supplementary Table 2. RNAi sequence used in this study**

| **siRNA** | **sense sequence** |
| --- | --- |
| miR-326 mimics | 5'- GACCUCCUUCCCGGGUCUCC-3' |
| miR-326 mimics negative  control | 5’-UCACAACCUCCUAGAAAGAGUAGA-3' |
| miR-326 inhibitor | 5'-GGAGACCCGGGAAGGAGGTC-3' |
| miR-326 inhibitor negative control | 5'-TCTACTCTTTCTAGGAGGTTGTGA-3' |
| hsa_circ_POLA2 -shRNA-1 | 5'-CACCGGTCATGACCTGTGCATATGCCGAAGCATATGCACAGGTCATGACC -3' |
| hsa_circ_POLA2 -shRNA-2 | 5'-CACCGCGGTGGCCTGGATAACATTTCGAAAAATGTTATCCAGGCCACCGC -3' |
| hsa_circ_POLA2 -shRNA-3 | 5'-CACCGGTGGCCTGGATAACATTTGCCGAAGCAAATGTTATCCAGGCCACC -3' |
| Negative control shRNA | 5'-CACCCGACGTGCACCACGTGCTACTCGTACTCTTGATGCCGAGCACGGAA-3' |

**Supplementary Table 3. Information on antibodies used in this study**

| **Antibody** | **WB** | **IHC** | **Specificity** | **Company** |
| --- | --- | --- | --- | --- |
| GAPDH | 1:5000 | / | Mouse monoclonal | Proteintech Group, China |
| GNB1 | 1:800 | 1:100 | Rabbit Polyclonal | Proteintech Group, China |
| p-ERK1/2 | / | 1:500 | Rabbit Polyclonal | Proteintech Group, China |
| ERK1/2 | 1:1000 | / | Rabbit Polyclonal | Proteintech Group Chicago, USA |
| AKT | 1:1000 | / | Rabbit Polyclonal | Proteintech Group Chicago, USA |
| p-ERK1/2 | 1:1000 | / | Rabbit Polyclonal | Proteintech Group Chicago, USA |
| Ki-67 | 1:1000 | 1:2000 | Rabbit Polyclonal | Proteintech Group Chicago, USA |
